# Prognostic Implications of Right Ventricular Function and Pulmonary Pressures Assessed by Echocardiography in Hospitalized Patients with COVID-19

**DOI:** 10.3390/jpm11121245

**Published:** 2021-11-24

**Authors:** Maria Vincenza Polito, Angelo Silverio, Marco Di Maio, Michele Bellino, Fernando Scudiero, Vincenzo Russo, Barbara Rasile, Carmine Alfano, Rodolfo Citro, Guido Parodi, Carmine Vecchione, Gennaro Galasso

**Affiliations:** 1 Division of Cardiology, Cardiovascular and Thoracic Department, San Giovanni di Dio e Ruggi d’Aragona University Hospital, 84125 Salerno, Italy; mvpolito@hotmail.it (M.V.P.); rodolfocitro@gmail.com (R.C.); 2 Department of Medicine, Surgery and Dentistry, University of Salerno, 84084 Salerno, Italy; marcodimaio88@gmail.com (M.D.M.); michelebellino8@gmail.com (M.B.); brasile@unisa.it (B.R.); calfano@unisa.it (C.A.); cvecchione@unisa.it (C.V.); ggalasso@unisa.it (G.G.); 3 Division of Cardiology, “Bolognini” Hospital, ASST Bergamo Est, 24068 Seriate, Italy; fscudiero@gmail.com; 4 Department of Translational Medical Sciences, University of Campania “Luigi Vanvitelli”–Monaldi and Cotugno Hospital, 80131 Naples, Italy; v.p.russo@libero.it; 5 Division of Interventional Cardiology, University Hospital of Sassari, 07100 Sassari, Italy; gparodi@uniss.it

**Keywords:** COVID-19, coronavirus, right ventricular dysfunction, TAPSE, pulmonary hypertension, RV–arterial coupling, outcome

## Abstract

Aims: Pulmonary involvement in Coronavirus disease 2019 (COVID-19) may affect right ventricular (RV) function and pulmonary pressures. The prognostic value of tricuspid annular plane systolic excursion (TAPSE), systolic pulmonary artery pressure (PAPS), and TAPSE/PAPS ratios have been poorly investigated in this clinical setting. Methods and results: This is a multicenter Italian study, including consecutive patients hospitalized for COVID-19. In-hospital mortality and pulmonary embolism (PE) were identified as the primary and secondary outcome measures, respectively. The study included 227 (16.1%) subjects (mean age 68 ± 13 years); intensive care unit (ICU) admission was reported in 32.2%. At competing risk analysis, after stratifying the population into tertiles, according to TAPSE, PAPS, and TAPSE/PAPS ratio values, patients in the lower TAPSE and TAPSE/PAPS tertiles, as well as those in the higher PAPS tertiles, showed a significantly higher incidence of death vs. the probability to be discharged during the hospitalization. At univariable logistic regression analysis, TAPSE, PAPS, and TAPSE/PAPS were significantly associated with a higher risk of death and PE, both in patients who were and were not admitted to ICU. At adjusted multivariable regression analysis, TAPSE, PAPS, and TAPSE/PAPS resulted in independently associated risk of in-hospital death (TAPSE: OR 0.85, CI 0.74–0.97; PAPS: OR 1.08, CI 1.03–1.13; TAPSE/PAPS: OR 0.02, CI 0.02 × 10^−1^–0.2) and PE (TAPSE: OR 0.7, CI 0.6–0.82; PAPS: OR 1.1, CI 1.05–1.14; TAPSE/PAPS: OR 0.02 × 10^−1^, CI 0.01 × 10^−2^–0.04). Conclusions: Echocardiographic evidence of RV systolic dysfunction, increased PAPS, and poor RV-arterial coupling may help to identify COVID-19 patients at higher risk of mortality and PE during hospitalization.

## 1. Introduction

Coronavirus disease 2019 (COVID-19) sparked in Wuhan (China) and spread to other countries, rapidly reaching the dimensions of pandemic [1]. COVID-19 has been associated with cardiovascular complications, including myocardial injury, arrhythmias, acute coronary syndromes, myocarditis, pericarditis, and heart failure (HF) [2,3]. The potential mechanisms involved include direct viral damage, cytokine storm, thrombocytosis, micro and macro thromboembolic events, diffuse intravascular coagulation, and hypoxemic vasoconstriction of the pulmonary circulation [4]. Given that COVID-19 involves the respiratory tract and may precipitate interstitial pneumonia, acute respiratory distress syndrome (ARDS), and pulmonary embolism (PE) [5], the effect on right ventricular (RV) function and pulmonary pressures are currently being investigated for the potential implications on patients treatment and outcome. Previous studies have shown that RV dysfunction [6] and pulmonary hypertension occur very frequently in patients with COVID-19, being reported in up to one-third of cases [7]. The RV, in contrast to the left ventricle (LV), is more susceptible to the increased afterload, related to pulmonary diseases [8]. Furthermore, vasopressors administration and mechanical ventilation may further contribute to the deterioration of RV function and pulmonary pressures in intensive care setting.

To date, few studies have investigated the effect of RV involvement and pulmonary hypertension in hospitalized patients with COVID-19. Thus, we aimed at evaluating routine echocardiographic assessment of RV function, pulmonary pressures, and RV-arterial coupling, Ref. [9] as well as their association with the occurrence of death and PE in patients hospitalized with COVID-19.

## 2. Methods

### 2.1. Study Design

This was a multicenter, retrospective observational study, including consecutive patients with confirmed diagnosis of COVID-19, admitted to seven Italian Hospitals (Bergamo, Naples, Sassari, and Salerno provinces) from 1 March to 22 April 2020. All cases were confirmed by real-time, reverse transcriptase—polymerase chain reaction analysis of throat swab specimens, performed in all patients at admission independently by symptoms; COVID-19 diagnosis was based on the World Health Organization criteria. At admission, all patients underwent medical history collection, physical examination, and laboratory evaluation. Chest X-ray and/or computed tomography (CT) scans were also performed to rule out pneumonia [10]. All patients included in the study were evaluated by the hospital cardiology service and underwent transthoracic echocardiography (TTE) within 48 h from the admission. This study was conducted according to the Declaration of Helsinki and approved by the institutional ethics committees. The need for individual informed consent was waived, due to the observational, retrospective design of the study.

### 2.2. Measures and Outcome

Baseline demographic, clinical, laboratory, and TTE data were collected and recorded on an electronic dedicated datasheet. In all patients, demographic (age, gender), clinical (comorbidities, symptoms at presentation, pharmacological therapy before and during hospitalization), and serum biomarkers (high-sensitivity troponin, D-dimer) at admission and echocardiographic data were collected, as well as information on patient clinical course (admission in intensive care unit (ICU) and necessity for respiratory support) and in-hospital complications (ARDS, acute myocardial injury, PE, acute HF), were registered. ARDS diagnosis was defined according to the Berlin definition [11].

Acute myocardial injury was defined as elevated cardiac troponin levels, with at least one value above the 99th percentile upper reference limit [12]. The diagnosis of PE was performed, according to the latest edition of ESC guidelines [13], and confirmed by computed tomography pulmonary angiography (CTPA). Acute HF was confirmed after clinical and echocardiographic evaluation according to the current guidelines [14].

At the time of the analysis, no patient was still hospitalized. The number of patients who died in the hospital, have recovered, and hospitalization length were also collected. In-hospital mortality was identified as the primary outcome of this study; PE was considered as the secondary outcome.

### 2.3. Transthoracic Echocardiography

TTE was performed, in accordance with the current recommendations [15]. Echocardiographic exam included the evaluation of left ventricular (LV), end-diastolic (EDV), and end-systolic volumes (ESV). LV ejection fraction (LVEF) was assessed using the modified Simpson’s rule in the apical two- and four-chamber view. Once optimized, RV visualization by probe adjustment, tricuspid annular plane systolic excursion (TAPSE) was calculated by aligning an M-mode cursor parallel with the RV free wall and entangling the tricuspid annulus. Pulmonary artery systolic pressure (PASP) was obtained through the tricuspid regurgitant jet velocity, using systolic trans-tricuspid pressure gradient calculated by the modified Bernoulli equation and adding the value of right atrial pressure, derived from the inferior vena cava diameter and degree of respiratory collapse [16]. RV dysfunction was defined, in accordance with the current guidelines [15], and PH through echocardiographic assessment, according to European Society of Cardiology (ESC) guidelines [17]. TAPSE/PASP ratio was calculated as a non-invasive index of RV-arterial coupling.

Mitral (MR) and tricuspid regurgitation (TR) were assessed using by the color doppler method [16]. Only patients with adequate echocardiographic windows and good quality echocardiographic images were included in this study.

### 2.4. Statistical Analysis

Categorical variables were reported as numbers and percentages. Distribution of continuous data were tested with the Kolmogorov–Smirnov and the Shapiro–Wilk test. Normally distributed variables were expressed as mean ± standard deviation (SD), whereas non-normal ones were expressed as median and interquartile range (IQR). The study population was divided into two groups, according to the clinical setting (ICU vs. non-ICU), and in tertiles, according to TAPSE, PASP, and TAPSE/PASP ratio values. Categorical variables between two groups (ICU vs. Non-ICU) were compared with chi-squared test or the Fisher exact test, when appropriate. Categorical variables between three groups (TTE tertiles) were compared with chi-squared tests. Continuous normally-distributed variables were compared between two groups by using the Student *t*-test and between three groups by using the one-way analysis of variance (ANOVA). Continuous non-normally-distributed variables were compared between two groups with the Mann–Withney test, and between three groups with the Kruskal–Wallis test.

The crude association between TAPSE, PASP, and TAPSE/PASP ratio values for the risk of the outcomes of interest was tested by using logistic regression models and presented as odds ratio (OR), with their 95% confidence intervals (CI). Receiver operating characteristic (ROC) curve analyses were performed to evaluate the discriminative performance of TAPSE, PASP, and TAPSE/PASP ratio for death and PE during the hospitalization.

We used the propensity score weighting technique to account for potential selection bias among patients with different TAPSE, PASP, and TAPSE/PASP ratio values. The propensity score model was developed by incorporating the clinical covariates potentially related to the exposure and/or outcome, regardless of their statistical significance or collinearity with other variables included in the model (non-parsimonious approach). The following baseline covariates were included in the propensity score model: male, age, hypertension, diabetes, dyslipidemia, smoke, coronary artery disease (CAD), prior myocardial infarction (MI), prior percutaneous coronary intervention (PCI), prior coronary artery bypass graft (CABG), pacemaker and/or implantable cardioverter-defibrillator (ICD) and/or cardiac resynchronization therapy (CRT), heart failure, history of atrial fibrillation (AF), previous stroke, chronic obstructive pulmonary disease (COPD), chronic kidney disease (CKD), and cancer. After propensity score weighting, standardized mean differences were calculated to assess the balance for all covariates included in the propensity score model. Values higher than 0.10 were considered statistically significant for differences among groups. Further multivariable adjustment for LVEF value was performed to adjust on the base of LV systolic dysfunction at the time of TTE examination.

A competing risk analysis for discharge free from death was performed and displayed by using Kaplan–Meier survival curves, stratified according to TAPSE, PASP, and TAPSE/PASP tertiles. The risk of the study outcome vs. the probability to be discharged was assessed by using the Log-Rank test.

For all tests, *p* value < 0.05 was considered statistically significant. Statistical analysis was performed by using SPSS software version 23.0 (SPSS Inc., Chicago, IL, USA) and R version 3.5.1 (R Foundation for Statistical Computing, Vienna, Austria).

## 3. Results

### 3.1. Study Population

A total of 1401 patients, with a confirmed diagnosis of COVID-19, were admitted to the participating centres; in-hospital mortality was reported of 12.9%. Out of the entire population, 227 (16.1%) subjects underwent TTE within 48 h from admission and were included in this analysis.

The characteristics of the overall population and of the study groups (ICU vs. non-ICU) are summarised in Table 1. The mean age was 68 ± 13 years and 62.6% of patients were male. At admission, most of patients presented with fever (154, 67.8%) and dyspnea (158, 69.6%); the median time between symptom onset and hospitalization was 6 days (IQR 2.5–10).

ICU admission was reported in 73 patients (32.2%; Table 1). Sixty-eight patients needed invasive-mechanical ventilation (IMV) (30%); non-invasive ventilation (NIV) was adopted in 44.1% of cases.

Patients admitted to ICU had higher prevalence of hypertension (71.2% vs. 56.5%, *p* = 0.047), dyspnea (87.7% vs. 61%, *p* < 0.001), and chest discomfort (45.2% vs. 23.4%, *p* = 0.001) at presentation. The number of days from symptoms onset to hospitalization were significantly lower in ICU group (4 vs. 6; *p* = 0.006). ICU group showed lower LVEF (51% vs. 56%, *p* < 0.001), lower TAPSE (20 vs. 21 mm, *p* < 0.001), higher LV ESV (50 vs. 46 mL, *p* = 0.007), and PASP (38 vs. 34 mmHg, *p* = 0.002) values than the non-ICU group.

Furthermore, the patients admitted to ICU had more frequently moderate-to-severe TR (34.2% vs. 14.9%, *p* = 0.002). Of note, ICU group required most frequently invasive (83.6 vs. 4.5%; *p* < 0.001) and non-invasive mechanical ventilation (61.6 vs. 35.7%; *p* < 0.001).

The study population was divided into tertiles, according to the TAPSE (Supplementary Table S1), PASP (Supplementary Table S2), and TAPSE/PASP ratios (Supplementary Table S3). Patients in the lower TAPSE and TAPSE/PASP ratio tertiles, as well as those in the higher PASP tertile, were the oldest and showed the highest prevalence of comorbidities, including CKD, COPD, and HF.

### 3.2. In-Hospital Clinical Outcomes

The median length of hospitalization was 16 days (IQR 10–27). In-hospital death occurred in 68 cases (30.1%) and PE in 32 (14.1%).

ICU group showed higher incidence of ARDS (82.2 vs. 30.5%; *p* < 0.001), acute cardiac injury (46.6 vs. 22.7%; *p* < 0.001), acute HF (34.2 vs. 9.1%; *p* < 0.001), and death (63.9 vs. 14.3%; *p* < 0.001), compared with non-ICU group.

Patients in the lower TAPSE and TAPSE/PASP ratio tertiles, and those in the higher PASP tertile, more frequently required IMV and ICU and more frequently experienced acute cardiac injury, acute HF, PE, and death during hospitalization (Supplementary Tables S1–S3).

At univariable logistic regression analysis, TAPSE, PASP, and TAPSE/PASP ratios were significantly associated with a higher risk of both death and PE, with a moderate discriminative performance (Table 2). This result was consistent in the subsets of patients admitted or not admitted to ICU with the exception of PASP, which was not significantly associated with the risk of PE in the non-ICU group (*p* = 0.064).

At propensity score weighted multivariable regression analysis (after multivariable adjustment for LVEF), TAPSE, PASP, and TAPSE/PASP resulted as independently associated with the risk of in-hospital death (Table 3). Moreover, all the multivariable regression models showed a good discriminative performance for the primary outcome. TAPSE, PASP, and TAPSE/PASP ratio were also independently associated with the risk to develop PE. LVEF was not significantly associated with PE, once adjusted for the PASP and TAPSE/PASP ratios.

**Figure 1:**
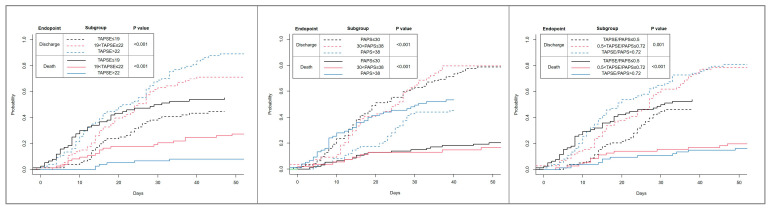
Kaplan–Meier survival curves for discharge free from death and in-hospital mortality, according to TAPSE, PASP, and TAPSE/PASP tertiles.

The risk of in-hospital death, according to the TAPSE, PASP, and TAPSE/PASP ratios, as well as the tertiles, were estimated, considering discharge alive as a competing risk (Figure 1). Lower TAPSE and TAPSE/PASP tertiles were significantly associated with poorer survival during the hospitalization (*p* < 0.001); higher PASP tertiles were also associated with a higher probability of in-hospital death.

## 4. Discussion

The main findings of this Italian multicenter observational study on hospitalized patients with COVID-19 can be summarized as follows:

(1) Conventional echocardiographic parameters, including TAPSE, PASP, and TAPSE/PASP ratios were independently associated with the risk of in-hospital death; this association was confirmed after adjusting for LV systolic function assessed by LVEF;
(2) TAPSE, PASP, and TAPSE/PASP were independently associated with the risk of PE, whereas LVEF did not show a significant association, independent from PASP and TAPSE/PASP values;
(3) At competing risk analysis, patients in the lowest TAPSE and TAPSE/PASP tertiles, as well as those in the highest PASP tertile, emerged as the groups with the highest risk of death during the hospitalization.

The present analysis is consistent with previous studies, showing a high probability of cardiovascular involvement in hospitalized patients with COVID-19, particularly in those admitted in ICU, as well as its detrimental effects of clinical status and in-hospital outcome [18,19,20,21,22]. This study included a very high-risk population, as suggested by the percentage of comorbidities, high prevalence of invasive and non-invasive ventilation, and high rate of cardiac complications. This risk profile may be partially attributed to the selective inclusion of patients who underwent TTE, based on clinical judgement; this study criterion may have contributed to the high percentage of mortality, registered in almost one-third of cases during the in-hospital course, which was substantially higher than that reported in previous studies [18,23].

Considering the pathophysiological hypothesis, for which COVID-19 induces lung damage and may acutely affect the RV and pulmonary pressures, we decided to evaluate if routinely used echocardiographic parameters, namely the TAPSE, PASP, and TAPSE/PASP ratios, which might have a prognostic role in hospitalized COVID-19 patients.

RV dysfunction can be attributed to different mechanisms: (1) systemic inflammation and hypoxemia inducing pulmonary vasoconstriction, (2) micro and/or macro thrombotic events affecting the pulmonary circulation, (3) the use of high-flow oxygen or mechanical ventilation therapy promoting increased RV afterload, (4) super-infection with other types of pneumonia, which should contribute to alteration of the pulmonary ventilo-perfusive unite, (5) the use of a-agonists (in case of hemodynamic instability), (6) elevated left atrial pressure, due to concomitant LV dysfunction and leading to elevated pulmonary pressures, (7) and a combination of the above. Regardless of its pathophysiology, the increase in RV afterload results in cardiac output reduction and hypotension, with consequent impaired coronary perfusion triggering a “snake biting its own tail” mechanism, for which RV dysfunction begets RV dysfunction [7,24,25,26]. Additionally, non-physiological transeptal pressure gradient between RV and LV may determine septal bowing, resulting in abnormal orientation of helical myofibrils and further reduction in LV cardiac function.

In the analysis of Kim et al. [27], RV dilation or dysfunction conferred a >2-fold increase in risk of in-hospital death and remained significant in multivariate analysis independently, by standard clinical- and biomarker-based assessment, confirming the prognostic utility of RV remodeling evaluation in COVID-19 patients.

Furthermore, in a small population of patients with COVID-19 pneumonia [28], those with cardiac injury showed RV dilatation, poorer pulmonary pressure, and TAPSE, compared with those without cardiac injury. Interestingly, the impaired RV function, assessed by the RV longitudinal strain (RVLS), has been associated with higher risk of mortality [29]. In our register, we collected more conventional TTE parameters, and we did not analyze RVLS, which was only seldomly reported. Indeed, speckle-tracking echocardiography is highly dependent of the images’ quality and may be challenging to use for patients admitted to ICU, who are on mechanical ventilation or are in supine or prone positions [30]. Speckle-tracking echocardiography also needs ECG-gating, adequate frame rate, and multiple cardiac cycles, acquired with similar heart rate. This may be difficult to perform in the pandemic clinical context and may expose sonographers to higher risk of infection.

In a previous study by our register, we have demonstrated that PE was a relatively common complication in hospitalized patients with COVID-19 and was associated with a poorer outcome [31]. Although associated with in-hospital mortality [32], in the present analysis, LVEF did not correlate with PE after adjustment for PAPS and TASE/PASP values, supporting the importance of these measures for PE risk stratification in this population.

In our population, non-contrast CT chest examinations were performed in all patients at admission and repeated, according to clinical judgment, for the evaluation of lung involvement by COVID-19.

CTPA was performed in patients with suspected PE. In patients with COVID-19, the discriminative ability of D-dimer for PE is substantially reduced, making this parameter inadequate for the assessment of PE pre-test probability [31]. In this scenario, the use of routine TTE parameters may be helpful for identifying patients with the highest probability of PE, who need further assessment by CTPA to confirm PE diagnosis and start timely anticoagulation therapy.

Anticoagulant and non-anticoagulant effects (anti-viral and anti-inflammatory) of heparin and synthetic heparin-like drugs have been well-established and advocated as potentially beneficial in reducing mortality in COVID-19 hospitalized patients, in which virus-induced coagulopathy is very common and multifactorial [33]. This benefit was particularly seen at prophylactic doses, in those with the highest D-Dimer values on admission, as well as the most severely ill patients. According to the current recommendations UFH or LMWH, used in our cohort in 82% patients, remains as the best choice of anticoagulant for all admitted COVID-19 patients and not only for those with thrombotic complications. However, the potential benefits of anticoagulation must be balanced against the risk of bleeding, and, at present, the optimal regimen remains to be determined [34].

Our study demonstrates the importance of TTE evaluation of RV and pulmonary pressure both in ICU and non-ICU patients, to stratify the risk of mortality. In our registry, patients in the lowest TAPSE and TAPSE/PASP tertile, and those in the highest PASP tertile, showed a higher probability to develop in-hospital complications and death [35]; this association was independent from LVEF, supporting the importance of this complementary information.

Moreover, the patients with RV systolic dysfunction also had significantly higher PASP values, as well as those with higher PASP, most frequently showed TAPSE impairment. This inverse correlation is also well-established in non-COVID-19 patients, supporting the susceptibility of RV to the afterload increase [36].

The present analysis highlights that a focused echocardiographic evaluation during hospitalization would be advisable in COVID-19 patients, in order to detect RV abnormalities and increased pulmonary pressures early. This assessment, made by conventional echocardiographic parameters, might play a key role in both critical and non-critical care setting for clinical management and identifying long-term cardiac sequelae of COVID-19.

## 5. Limitations

Our results should be interpreted in light of the limitations related to the retrospective observational design of the study. Although we reviewed all consecutive patients who were infected by SARS-CoV-2 and admitted to different institutions throughout Italian Country, TTE was performed in only one-sixth of the entire patient population. We included (in the analysis) only the patients evaluated by cardiologists and for whom good echocardiographic windows and quality of TTE images were available. The need of good quality echocardiographic data restricted our analysis to 227 cases, which cannot be representative of the entire COVID-19 population. Furthermore, the use of TTE in only a limited percentage of patients was probably reserved to more challenging cases, selecting a subset of patients at higher risk; additionally, pulse oximetry data, at the time of echocardiographic examinations, were not collected.

Parameters other than TAPSE, such as fractional area change, peak systolic velocity (S’), and especially the RV strain, have not been assessed in this study but we cannot exclude their potential utility in this patient’s setting.

Owing to the absence of TTE data before hospitalization, we cannot exclude the presence of preexistent LV and/or RV impairment in analyzed patients. However, our aim was not to explore the prognostic role of new-onset RV dysfunction, with or without increased pulmonary pressures, but to investigate the association between TAPSE, PASP, and TAPSE/PASP ratios, evaluated within 48 h from admission, as well as mortality or PE during the hospitalization in COVID-19 patients.

Lastly, we were not able to analyze the impact of the different experimental COVID-19 therapies on clinical outcome, the potential changes after specific treatment, and their role in follow-up.

Certainly, larger prospective studies are required to confirm our preliminary findings and to evaluate the aspects that have not been addressed by this study.

## 6. Conclusions

RV systolic dysfunction, high pulmonary pressures, and poor RV-arterial coupling independently predict the risk of mortality and PE in hospitalized patients with COVID-19, both in the ICU and ward. The implementation of a comprehensive TTE assessment, at hospital admission, may help clinical decision-making and prognostic stratification in hospitalized patients with COVID-19.

## Figures and Tables

**Table 1:** Baseline characteristics of the study population according to the admission or not in ICU.

	Overall	No ICU	ICU	*p*
Patients, *n*	227	154	73	
**Demographics**				
Female gender, *n* (%)	85 (37.4)	62 (40.3)	23 (31.5)	0.260
Male gender, *n* (%)	142 (62.6)	92 (59.7)	50 (68.5)	0.260
Age, years	70.00 [60.00, 79.00]	71.00 [60.00, 81.00]	69.00 [60.00, 76.00]	0.164
**Medical history**				
Smoker, *n* (%)	42 (18.5)	25 (16.2)	17 (23.3)	0.273
Hypertension, *n* (%)	139 (61.2)	87 (56.5)	52 (71.2)	0.047
Diabetes, *n* (%)	64 (28.2)	41 (26.6)	23 (31.5)	0.545
Dyslipidaemia, *n* (%) *	62 (30.7)	40 (29.2)	22 (33.8)	0.613
CKD, *n* (%)	45 (19.8)	28 (18.2)	17 (23.3)	0.470
COPD, *n* (%)	46 (20.3)	34 (22.1)	12 (16.4)	0.418
Cancer, *n* (%)	27 (11.9)	17 (11.0)	10 (13.7)	0.720
History of AF, *n* (%) **	46 (20.4)	33 (21.6)	13 (17.8)	0.631
Previous Stroke, *n* (%)	18 (7.9)	14 (9.1)	4 (5.5)	0.498
Heart Failure, *n* (%)	22 (9.7)	14 (9.1)	8 (11.0)	0.838
CAD, *n* (%)	35 (15.4)	22 (14.3)	13 (17.8)	0.624
Prior MI, *n* (%)	37 (16.3)	22 (14.3)	15 (20.5)	0.317
Prior PCI, *n* (%)	36 (15.9)	23 (14.9)	13 (17.8)	0.720
Prior CABG, *n* (%)	13 (5.7)	7 (4.5)	6 (8.2)	0.420
PM/ICD/CRT, *n* (%)	9 (4.0)	5 (3.2)	4 (5.5)	0.659
**Symptoms at presentation**				
Fever, *n* (%)	154 (67.8)	102 (66.2)	52 (71.2)	0.548
Dyspnoea, *n* (%)	158 (69.6)	94 (61.0)	64 (87.7)	<0.001
Cough, *n* (%)	87 (38.3)	58 (37.7)	29 (39.7)	0.879
Chest discomfort, *n* (%)	69 (30.4)	36 (23.4)	33 (45.2)	0.001
GI symptoms, *n* (%)	30 (13.2)	22 (14.3)	8 (11.0)	0.630
Symptoms onset tohospitalization, days	6.00 [2.50, 10.00]	6.00 [3.00, 10.00]	4.00 [1.00, 7.00]	0.006
**Pharmacological therapy at admission**				
ACEi or ARB, *n* (%)	99 (43.6)	56 (36.4)	43 (58.9)	0.002
Betablocker, *n* (%)	59 (26.0)	41 (26.6)	18 (24.7)	0.878
Diuretic, *n* (%)	47 (20.7)	26 (16.9)	21 (28.8)	0.059
P2Y12 inhibitor, *n* (%)	21 (9.3)	14 (9.1)	7 (9.6)	1.000
ASA, *n* (%)	67 (29.5)	44 (28.6)	23 (31.5)	0.766
Statin, *n* (%)	71 (31.3)	43 (27.9)	28 (38.4)	0.153
Insulin, *n* (%)	32 (14.1)	19 (12.3)	13 (17.8)	0.367
VKA or NOAC, *n* (%)	42 (18.5)	29 (18.8)	13 (17.8)	0.998
**Serum biomarkers**				
Troponin hs, n 99thpercentile; peak ^∞^	24.40 [2.78, 225.00]	22.10 [5.15, 215.50]	43.70 [1.80, 248.00]	0.718
D-dimer, peak; ng/mL ^¥^	625.00 [100.75, 1994.00]	564.00 [176.00, 1397.50]	1363.50 [13.13, 2735.00]	0.173
**Echocardiographic data**				
LVEF, %	55.00 [50.00, 59.00]	56.00 [50.00, 60.00]	51.00 [45.00, 55.00]	<0.001
LVEDV, mL	103.00 [89.00, 120.00]	101.00 [88.00, 119.00]	103.00 [90.50, 130.25]	0.271
LVESV, mL	47.00 [39.00, 58.10]	46.00 [38.00, 53.00]	50.00 [43.00, 60.60]	0.007
TAPSE, mm	21.00 [18.00, 23.00]	21.00 [19.00, 24.00]	20.00 [16.00, 21.00]	<0.001
PASP, mmHg	33.00 [30.00, 40.00]	32.00 [29.00, 40.00]	36.00 [30.00, 45.00]	0.002
Moderate or severe MR, *n* (%)	36 (15.9)	23 (14.9)	13 (17.8)	0.720
Moderate or severe TR, *n* (%)	48 (21.1)	23 (14.9)	25 (34.2)	0.002
**SARS-COV 2 therapies**				
Glucocorticoid, *n* (%)	102 (44.9)	63 (40.9)	39 (53.4)	0.104
Antiviral, *n* (%)	119 (52.4)	66 (42.9)	53 (72.6)	<0.001
Antibiotics, *n* (%)	167 (73.6)	103 (66.9)	64 (87.7)	0.002
Tocilizumab, *n* (%) ^#^	1 (1.0)	1 (1.4)	0 (0.0)	1.000
Hydroxychloroquine, *n* (%)	181 (79.7)	116 (75.3)	65 (89.0)	0.026
UFH or LMWH, *n* (%) ^§^	184 (81.8)	117 (77.0)	67 (91.8)	0.012
**In hospital data and complications**				
IMV, *n* (%)	68 (30.0)	7 (4.5)	61 (83.6)	<0.001
NIV, *n* (%)	100 (44.1)	55 (35.7)	45 (61.6)	<0.001
ARDS, *n* (%)	107 (47.1)	47 (30.5)	60 (82.2)	<0.001
Acute cardiac injury, *n* (%)	69 (30.4)	35 (22.7)	34 (46.6)	<0.001
Pulmonary embolism, *n* (%)	32 (14.1)	21 (13.6)	11 (15.1)	0.932
Acute HF, *n* (%)	39 (17.2)	14 (9.1)	25 (34.2)	<0.001
Death, *n* (%)	68 (30.1)	22 (14.3)	46 (63.9)	<0.001
Hospitalization, days	16.00 [10.00, 27.00]	18.00 [12.00, 27.00]	15.00 [7.00, 28.00]	0.392

* Available in 202 of 227 patients; ** available in 226 of 227 patients; ^#^ available in 102 of 227 patients; ^§^ available in 225 of 227 patients; ^∞^ available in 113 of 227 patients; ¥ available in 114 of 227 patients. Categorical variables are presented as numbers (%). Continuous, non-normally distributed variables are presented as median (interquartile range-IQR). CKD, chronic kidney disease; COPD, chronic obstructive pulmonary disease; AF, atrial fibrillation; CAD, coronary artery disease; MI, myocardial infarction; PCI, percutaneous coronary intervention; CABG, coronary artery bypass graft; PM, pacemaker; ICD, implantable cardioverter-defibrillator; CRT, cardiac resynchronization therapy; GI, gastrointestinal; ACE-I, angiotensin-converting enzyme inhibitor; ARB, angiotensin receptor blocker; ASA, aspirin; VKA, vitamin K oral anticoagulant; NOAC, non-vitamin K oral anticoagulant; LVEF, left ventricular ejection fraction; LVEDV, left ventricular end diastolic volume; ESV, left ventricular end systolic volume; TAPSE, tricuspid annular plane systolic excursion; PASP, systolic pulmonary artery pressure; MR, mitral regurgitation; TR, tricuspid regurgitation; UFH, unfractionated heparin; LMWH, low molecular weight heparin; ICU, intensive care unit, IMV, invasive mechanical ventilation; NIV, non invasive ventilation; ARDS, acute respiratory distress syndrome; HF, heart failure.

**Table 2:** Univariable logistic regression analysis.

			OR (CI)	*p*	AUC
**Overall**	Death	TAPSE	0.75 (0.68, 0.82)	<0.001	0.772
		PASP	1.09 (1.06, 1.13)	<0.001	0.724
		TAPSE/PASP	0.05 × 10^−1^ (0.08 × 10^−2^, 0.03)	<0.001	0.770
	Pulmonary Embolism	TAPSE	0.8 (0.72, 0.88)	<0.001	0.739
		PASP	1.08 (1.04, 1.12)	<0.001	0.703
		TAPSE/PASP	0.01 (0.01 × 10^−1^, 0.09)	<0.001	0.736
**ICU**	Death	TAPSE	0.79 (0.68, 0.92)	0.003	0.714
		PASP	1.09 (1.02, 1.15)	0.006	0.765
		TAPSE/PASP	0.01 (0.08 × 10^−2^, 0.17)	<0.001	0.770
	Pulmonary Embolism	TAPSE	0.71 (0.58, 0.87)	0.001	0.720
		PASP	1.15 (1.06, 1.26)	0.002	0.654
		TAPSE/PASP	0.03 × 10^−2^ (0.02 × 10^−4^, 0.06)	0.002	0.704
**No ICU**	Death	TAPSE	0.74 (0.65, 0.85)	<0.001	0.720
		PASP	1.09 (1.04, 1.45)	<0.001	0.724
		TAPSE/PASP	0.03 × 10^−1^ (0.02 × 10^−2^, 0.05)	<0.001	0.750
	Pulmonary Embolism	TAPSE	0.82 (0.72, 0.92)	<0.001	0.817
		PASP	1.05 (1, 1.1)	0.064	0.812
		TAPSE/PASP	0.02 (0.02 × 10^−1^, 0.28)	0.003	0.831

Univariable logistic regression analysis for the three echocardiographic parameters describing the RV systolic function (TAPSE), the PA systolic pressure (PASP), and RV-PA coupling (TAPSE/PASP) against the two endpoints (death and pulmonary embolism) in the overall population, ICU subgroup, and patients not admitted to the ICU. TAPSE, tricuspid annular plane systolic excursion; PASP, systolic pulmonary artery pressure; ICU, intensive care unit.

**Table 3:** Weighted multivariable logistic regression analysis.

		OR (CI)	*p*	AUC
**Death**	TAPSE	0.85 (0.74, 0.97)	0.017	0.820
	EF	0.92 (0.88, 0.97)	0.001	
	PASP	1.08 (1.03, 1.13)	0.002	0.790
	EF	0.91 (0.87, 0.95)	<0.001	
	TAPSE/PASP	0.02 (0.02 × 10^−1^, 0.2)	<0.001	0.810
	EF	0.93 (0.89, 0.97)	0.001	
**Pulmonary Embolism**	TAPSE	0.7 (0.6, 0.82)	<0.001	0.690
	EF	1.07 (1, 1.15)	0.041	
	PASP	1.1 (1.05, 1.14)	<0.001	0.700
	EF	1.01 (0.96, 1.07)	0.671	
	TAPSE/PASP	0.02 × 10^−1^ (0.01 × 10^−2^, 0.04)	<0.001	0.720
	EF	1.05 (0.99, 1.11)	0.132	

Weighted multivariable logistic regression models, analyzing the three echocardiographic parameters describing the RV systolic function (TAPSE), PA systolic pressure (PASP), and RV-PA coupling (TAPSE/PASP), with EF as a second covariate against the two endpoints (death and pulmonary embolism) in the overall population. Three different propensity weighting models were applied for TAPSE, PASP, and TAPSE/PASP variables. TAPSE, tricuspid annular plane systolic excursion; PASP, systolic pulmonary artery pressure; EF, ejection fraction.

## Data Availability

Not applicable.

## Supplementary Materials

The following are available online at https://www.mdpi.com/article/10.3390/jpm11121245/s1, Table S1: Characteristics of the study population stratified by TAPSE tertiles, Table S2: Characteristics of the study population stratified by PASP tertiles, Table S3: Characteristics of the study population stratified by TAPSE/PASP tertiles.
