# Supplementary material for: Prognostic Implications of Right Ventricular Function and Pulmonary Pressures Assessed by Echocardiography in Hospitalized Patients with COVID-19
Source: J Pers Med. 2021 Nov 24;11(12):1245. doi: 10.3390/jpm11121245 (PMC8705674; doi:10.3390/jpm11121245)
Supplement: Supplementary file 1 [file jpm-11-01245-s001.zip › jpm-1471342-supplementary.pdf]

Supplementary Materials:

Table S1. Characteristics of the study population stratified by TAPSE tertiles.

	**TAPSE≤19**	**19<TAPSE≤22**	**TAPSE>22**	**p**
Patients, n	77	75	75	
**Demographics**				
Male gender, n (%)	46 (59.7)	53 (70.7)	43 (57.3)	0.198
Age, years	76.00 [67.00, 83.00]	69.00 [60.00, 77.00]	65.00 [54.50, 74.50]	<0.001
**Medical history**				
Smoker, n (%)	24 (31.2)	8 (10.7)	10 (13.3)	0.002
Hypertension, n (%)	53 (68.8)	47 (62.7)	39 (52.0)	0.099
Diabetes, n (%)	27 (35.1)	21 (28.0)	16 (21.3)	0.170
Dyslipidaemia, n (%)*	24 (34.3)	22 (29.7)	16 (27.6)	0.698
CKD, n (%)	27 (35.1)	8 (10.7)	10 (13.3)	<0.001
COPD, n (%)	26 (33.8)	9 (12.0)	11 (14.7)	0.001
Cancer, n (%)	6 (7.8)	11 (14.7)	10 (13.3)	0.380
History of AF, n (%)**	19 (24.7)	13 (17.6)	14 (18.7)	0.503
Previous Stroke, n (%)	9 (11.7)	6 (8.0)	3 (4.0)	0.215
Heart Failure, n (%)	14 (18.2)	6 (8.0)	2 (2.7)	0.004
CAD, n (%)	17 (22.1)	10 (13.3)	8 (10.7)	0.124
Prior MI, n (%)	20 (26.0)	10 (13.3)	7 (9.3)	0.015
Prior PCI, n (%)	18 (23.4)	12 (16.0)	6 (8.0)	0.034
Prior CABG, n (%)	8 (10.4)	4 (5.3)	1 (1.3)	0.055
PM/ICD/CRT, n (%)	3 (3.9)	5 (6.7)	1 (1.3)	0.246
**Symptoms at presentation**				
Fever, n (%)	58 (75.3)	45 (60.0)	51 (68.0)	0.129
Dyspnoea, n (%)	54 (70.1)	59 (78.7)	45 (60.0)	0.045
Cough, n (%)	34 (44.2)	23 (30.7)	30 (40.0)	0.217
Chest discomfort, n (%)	31 (40.3)	21 (28.0)	17 (22.7)	0.053
GI symptoms, n (%)	13 (16.9)	7 (9.3)	10 (13.3)	0.389
Symptoms onset to hospitalization, days	4.00 [2.00, 7.00]	6.00 [1.00, 10.00]	7.00 [4.00, 10.00]	0.001
**Pharmacological therapy at hospitalization**				
ACEi or ARB, n (%)	42 (54.5)	33 (44.0)	24 (32.0)	0.020
Betablocker, n (%)	22 (28.6)	16 (21.3)	21 (28.0)	0.530
Diuretic, n (%)	28 (36.4)	8 (10.7)	11 (14.7)	<0.001
P2Y12 inhibitor, n (%)	13 (16.9)	4 (5.3)	4 (5.3)	0.018
ASA, n (%)	29 (37.7)	22 (29.3)	16 (21.3)	0.088

Statin, n (%)	34 (44.2)	21 (28.0)	16 (21.3)	0.008
Insulin, n (%)	16 (20.8)	12 (16.0)	4 (5.3)	0.020
VKA or NOAC, n (%)	17 (22.1)	13 (17.3)	12 (16.0)	0.597
**Serum biomarkers**				
Troponin hs, n · 99th percentile; peak ∞	80.00 [21.40, 390.00]	6.86 [0.11, 85.46]	21.75 [7.43, 42.78]	0.002
D-dimer, peak; ng/ml ¥	1262.00 [417.50, 2462.50]	35.20 [1.69, 510.00]	928.00 [459.00, 1888.00]	<0.001
**Echocardiographic data**				
LVEF, %	50.00 [40.00, 55.00]	55.00 [50.00, 58.00]	57.00 [55.00, 60.00]	<0.001
LVEDV, mL	99.00 [88.00, 117.00]	110.00 [92.00, 124.00]	98.00 [88.50, 118.50]	0.203
LVESV, mL	48.00 [41.00, 60.30]	50.00 [40.00, 60.15]	44.00 [38.00, 51.00]	0.031
TAPSE, mm	17.00 [14.00, 18.00]	21.00 [20.00, 21.00]	24.00 [23.00, 26.00]	<0.001
PASP, mmHg	40.00 [35.00, 47.00]	30.00 [30.00, 38.00]	30.00 [28.00, 35.00]	<0.001
Moderate or severe MR, n (%)	20 (26.0)	4 (5.3)	12 (16.0)	0.002
Moderate or severe TR, n (%)	32 (41.6)	5 (6.7)	11 (14.7)	<0.001
**SARS-COV 2 therapies**				
Glucocorticoid, n (%)	40 (51.9)	24 (32.0)	38 (50.7)	0.022
Antiviral, n (%)	42 (54.5)	36 (48.0)	41 (54.7)	0.644
Antibiotics, n (%)	59 (76.6)	46 (61.3)	62 (82.7)	0.009
Tocilizumab, n (%) #	0 (0.0)	0 (0.0)	1 (2.3)	0.514
Hydroxycloroquine, n (%)	60 (77.9)	56 (74.7)	65 (86.7)	0.167
UFH or LMWH, n (%) §	61 (79.2)	55 (75.3)	68 (90.7)	0.042
**In hospital data and complications**				
ICU, n (%)	36 (46.8)	24 (32.0)	13 (17.3)	0.001
IMV, n (%)	32 (41.6)	24 (32.0)	12 (16.0)	0.002
NIV, n (%)	46 (59.7)	28 (37.3)	26 (34.7)	0.003
ARDS, n (%)	42 (54.5)	35 (46.7)	30 (40.0)	0.198
Acute cardiac injury, n (%)	39 (50.6)	18 (24.0)	12 (16.0)	<0.001
Pulmonary embolism, n (%)	20 (26.0)	9 (12.0)	3 (4.0)	<0.001
Acute HF, n (%)	30 (39.0)	7 (9.3)	2 (2.7)	<0.001
Death, n (%)	42 (54.5)	20 (27.0)	6 (8.0)	<0.001
Hospitalization, days	15.00 [8.00, 24.00]	18.00 [12.00, 28.00]	19.00 [10.50, 30.50]	0.016

Table S2. Characteristics of the study population stratified by PASP tertiles.

	PASP≤30	30<PASP≤38	PASP>38	p
Patients, n	98	54	75	
**Demographics**				
Male gender, n (%)	68 (69.4)	32 (59.3)	42 (56.0)	0.167
Age, years	63.50 [57.25, 74.75]	73.00 [65.25, 79.00]	75.00 [62.00, 81.00]	0.001
**Medical history**				
Smoker, n (%)	17 (17.3)	10 (18.5)	15 (20.0)	0.906
Hypertension, n (%)	53 (54.1)	39 (72.2)	47 (62.7)	0.085
Diabetes, n (%)	27 (27.6)	12 (22.2)	25 (33.3)	0.377
Dyslipidaemia, n (%)*	25 (29.1)	16 (33.3)	21 (30.9)	0.876
CKD, n (%)	6 (6.1)	13 (24.1)	26 (34.7)	<0.001
COPD, n (%)	14 (14.3)	10 (18.5)	22 (29.3)	0.048
Cancer, n (%)	15 (15.3)	3 (5.6)	9 (12.0)	0.206
History of AF, n (%)**	13 (13.3)	14 (26.4)	19 (25.3)	0.068
Previous Stroke, n (%)	6 (6.1)	8 (14.8)	4 (5.3)	0.098
Heart Failure, n (%)	3 (3.1)	5 (9.3)	14 (18.7)	0.003
CAD, n (%)	13 (13.3)	8 (14.8)	14 (18.7)	0.616
Prior MI, n (%)	11 (11.2)	11 (20.4)	15 (20.0)	0.196
Prior PCI, n (%)	13 (13.3)	8 (14.8)	15 (20.0)	0.472
Prior CABG, n (%)	3 (3.1)	3 (5.6)	7 (9.3)	0.212
PM/ICD/CRT, n (%)	2 (2.0)	4 (7.4)	3 (4.0)	0.268
**Symptoms at presentation**				
Fever, n (%)	61 (62.2)	39 (72.2)	54 (72.0)	0.290
Dyspnoea, n (%)	69 (70.4)	39 (72.2)	50 (66.7)	0.775
Cough, n (%)	35 (35.7)	17 (31.5)	35 (46.7)	0.169
Chest discomfort, n (%)	28 (28.6)	13 (24.1)	28 (37.3)	0.237
GI symptoms, n (%)	8 (8.2)	7 (13.0)	15 (20.0)	0.074
Symptoms onset to hospitalization, days	7.00 [1.00, 10.00]	5.00 [2.00, 10.00]	5.50 [3.00, 9.25]	0.719
**Pharmacological therapy at hospitalization**				
ACEi or ARB, n (%)	37 (37.8)	24 (44.4)	38 (50.7)	0.235
Betablocker, n (%)	18 (18.4)	21 (38.9)	20 (26.7)	0.022
Diuretic, n (%)	11 (11.2)	9 (16.7)	27 (36.0)	<0.001
P2Y12 inhibitor, n (%)	5 (5.1)	5 (9.3)	11 (14.7)	0.099
ASA, n (%)	25 (25.5)	18 (33.3)	24 (32.0)	0.507
Statin, n (%)	27 (27.6)	16 (29.6)	28 (37.3)	0.371
Insulin, n (%)	11 (11.2)	9 (16.7)	12 (16.0)	0.553

VKA or NOAC, n (%)	12 (12.2)	13 (24.1)	17 (22.7)	0.104
**Serum biomarkers**				
Troponin hs, n · 99th percentile; peak ∞	6.86 [0.18, 22.25]	33.00 [10.72, 238.75]	150.00 [47.00, 475.00]	<0.001
D-dimer, peak; ng/ml ¥	56.00 [2.78, 894.25]	568.00 [252.50, 1414.50]	1500.00 [518.25, 3139.75]	<0.001
**Echocardiographic data**				
LVEF, %	55.00 [50.25, 60.00]	55.00 [50.00, 58.75]	51.00 [44.50, 58.00]	0.005
LVEDV, mL	108.00 [90.00, 122.00]	97.00 [90.00, 119.00]	99.00 [85.00, 116.00]	0.137
LVESV, mL	47.50 [40.00, 59.00]	48.00 [41.00, 53.00]	46.00 [37.00, 60.00]	0.920
TAPSE, mm	22.00 [20.00, 24.00]	20.50 [18.25, 23.00]	18.00 [14.50, 20.50]	<0.001
PASP, mmHg	29.00 [25.00, 30.00]	35.00 [32.00, 37.00]	45.00 [40.00, 50.00]	<0.001
Moderate or severe MR, n (%)	2 (2.0)	12 (22.2)	22 (29.3)	<0.001
Moderate or severe TR, n (%)	0 (0.0)	9 (16.7)	39 (52.0)	<0.001
**SARS-COV 2 therapies**				
Glucocorticoid, n (%)	43 (43.9)	22 (40.7)	37 (49.3)	0.602
Antiviral, n (%)	57 (58.2)	25 (46.3)	37 (49.3)	0.302
Antibiotics, n (%)	75 (76.5)	35 (64.8)	57 (76.0)	0.247
Tocilizumab, n (%) #	1 (1.8)	0 (0.0)	0 (0.0)	0.671
Hydroxycloroquine, n (%)	84 (85.7)	40 (74.1)	57 (76.0)	0.143
UFH or LMWH, n (%) §	82 (84.5)	43 (79.6)	59 (79.7)	0.647
**In hospital data and complications**				
ICU, n (%)	28 (28.6)	12 (22.2)	33 (44.0)	0.020
IMV, n (%)	26 (26.5)	9 (16.7)	33 (44.0)	0.002
NIV, n (%)	39 (39.8)	20 (37.0)	41 (54.7)	0.073
ARDS, n (%)	46 (46.9)	18 (33.3)	43 (57.3)	0.027
Acute cardiac injury, n (%)	21 (21.4)	12 (22.2)	36 (48.0)	<0.001
Pulmonary embolism, n (%)	7 (7.1)	6 (11.1)	19 (25.3)	0.002
Acute HF, n (%)	8 (8.2)	6 (11.1)	25 (33.3)	<0.001
Death, n (%)	19 (19.6)	9 (16.7)	40 (53.3)	<0.001
Hospitalization, days	16.50 [10.00, 32.00]	16.50 [12.00, 29.00]	15.00 [8.00, 25.00]	0.107

Table S3. Characteristics of the study population stratified by TAPSE/PASP tertiles.

	TAPSE/PASP ≤0.5	0.5 <TAPSE/PASP≤ 0.72	TAPSE/PASP >0.72	p
Patients, n	78	73	76	
**Demographics**				
Male gender, n (%)	45 (57.7)	47 (64.4)	50 (65.8)	0.540
Age, years	75.00 [62.75, 80.00]	72.00 [64.00, 80.00]	62.00 [55.00, 73.00]	<0.001
**Medical history**				
Smoker, n (%)	22 (28.2)	8 (11.0)	12 (15.8)	0.018
Hypertension, n (%)	53 (67.9)	46 (63.0)	40 (52.6)	0.139
Diabetes, n (%)	24 (30.8)	22 (30.1)	18 (23.7)	0.561
Dyslipidaemia, n (%)*	23 (32.4)	25 (36.2)	14 (22.6)	0.222
CKD, n (%)	27 (34.6)	13 (17.8)	5 (6.6)	<0.001
COPD, n (%)	25 (32.1)	11 (15.1)	10 (13.2)	0.006
Cancer, n (%)	8 (10.3)	9 (12.3)	10 (13.2)	0.848
History of AF, n (%)**	20 (25.6)	14 (19.4)	12 (15.8)	0.308
Previous Stroke, n (%)	6 (7.7)	8 (11.0)	4 (5.3)	0.435
Heart Failure, n (%)	15 (19.2)	5 (6.8)	2 (2.6)	0.001
CAD, n (%)	16 (20.5)	9 (12.3)	10 (13.2)	0.304
Prior MI, n (%)	18 (23.1)	12 (16.4)	7 (9.2)	0.066
Prior PCI, n (%)	16 (20.5)	13 (17.8)	7 (9.2)	0.136
Prior CABG, n (%)	7 (9.0)	4 (5.5)	2 (2.6)	0.237
PM/ICD/CRT, n (%)	4 (5.1)	4 (5.5)	1 (1.3)	0.347
**Symptoms at presentation**				
Fever, n (%)	62 (79.5)	42 (57.5)	50 (65.8)	0.014
Dyspnoea, n (%)	54 (69.2)	55 (75.3)	49 (64.5)	0.352
Cough, n (%)	37 (47.4)	20 (27.4)	30 (39.5)	0.039
Chest discomfort, n (%)	28 (35.9)	22 (30.1)	19 (25.0)	0.339
GI symptoms, n (%)	17 (21.8)	5 (6.8)	8 (10.5)	0.018
Symptoms onset to hospitalization, days	5.00 [3.00, 7.00]	6.00 [1.00, 10.00]	7.00 [3.00, 10.00]	0.312
**Pharmacological therapy at hospitalization**				
ACEi or ARB, n (%)	45 (57.7)	29 (39.7)	25 (32.9)	0.006
Betablocker, n (%)	25 (32.1)	19 (26.0)	15 (19.7)	0.219
Diuretic, n (%)	30 (38.5)	8 (11.0)	9 (11.8)	<0.001
P2Y12 inhibitor, n (%)	12 (15.4)	5 (6.8)	4 (5.3)	0.066
ASA, n (%)	26 (33.3)	26 (35.6)	15 (19.7)	0.069
Statin, n (%)	33 (42.3)	22 (30.1)	16 (21.1)	0.017
Insulin, n (%)	14 (17.9)	14 (19.2)	4 (5.3)	0.025

VKA or NOAC, n (%)	19 (24.4)	13 (17.8)	10 (13.2)	0.198
**Serum biomarkers**				
Troponin hs, n · 99th percentile; peak ∞	150.00 [42.45, 525.00]	15.40 [0.15, 69.94]	9.00 [2.10, 23.00]	<0.001
D-dimer, peak; ng/ml ¥	1616.00 [564.75, 2605.00]	106.50 [4.30, 621.00]	549.50 [31.30, 1681.75]	<0.001
**Echocardiographic data**				
LVEF, %	50.00 [43.25, 55.75]	55.00 [50.00, 58.00]	56.00 [55.00, 60.00]	<0.001
LVEDV, mL	96.00 [85.00, 116.00]	105.00 [89.00, 127.00]	108.00 [92.50, 120.00]	0.163
LVESV, mL	46.00 [39.00, 60.00]	49.25 [39.00, 60.00]	46.00 [40.00, 53.00]	0.504
TAPSE, mm	17.00 [14.25, 19.00]	21.00 [20.00, 23.00]	24.00 [22.00, 26.00]	<0.001
PASP, mmHg	44.50 [40.00, 50.00]	32.00 [30.00, 37.00]	28.00 [25.00, 30.00]	<0.001
Moderate or severe MR, n (%)	21 (26.9)	9 (12.3)	6 (7.9)	0.003
Moderate or severe TR, n (%)	37 (47.4)	9 (12.3)	2 (2.6)	<0.001
**SARS-COV 2 therapies**				
Glucocorticoid, n (%)	41 (52.6)	24 (32.9)	37 (48.7)	0.038
Antiviral, n (%)	37 (47.4)	37 (50.7)	45 (59.2)	0.321
Antibiotics, n (%)	58 (74.4)	50 (68.5)	59 (77.6)	0.441
Tocilizumab, n (%) #	0 (0.0)	0 (0.0)	1 (2.3)	0.514
Hydroxycloroquine, n (%)	57 (73.1)	59 (80.8)	65 (85.5)	0.152
UFH or LMWH, n (%) §	60 (77.9)	60 (82.2)	64 (85.3)	0.493
**In hospital data and complications**				
ICU, n (%)	35 (44.9)	19 (26.0)	19 (25.0)	0.012
IMV, n (%)	32 (41.0)	18 (24.7)	18 (23.7)	0.031
NIV, n (%)	44 (56.4)	30 (41.1)	26 (34.2)	0.018
ARDS, n (%)	38 (48.7)	38 (52.1)	31 (40.8)	0.365
Acute cardiac injury, n (%)	37 (47.4)	19 (26.0)	13 (17.1)	<0.001
Pulmonary embolism, n (%)	21 (26.9)	7 (9.6)	4 (5.3)	<0.001
Acute HF, n (%)	25 (32.1)	12 (16.4)	2 (2.6)	<0.001
Death, n (%)	42 (53.8)	14 (19.4)	12 (15.8)	<0.001
Hospitalization, days	15.00 [8.00, 24.00]	19.00 [13.00, 30.00]	16.00 [10.00, 31.50]	0.012
